# Radiomic Signatures for Predicting *EGFR* Mutation Status in Lung Cancer Brain Metastases

**DOI:** 10.3389/fonc.2022.931812

**Published:** 2022-07-14

**Authors:** Lie Zheng, Hui Xie, Xiao Luo, Yadi Yang, Yijun Zhang, Yue Li, Shaohan Yin, Hui Li, Chuanmiao Xie

**Affiliations:** ^1^ State Key Laboratory of Oncology in South China, Collaborative Innovation Center for Cancer Medicine, Sun Yat-sen University Cancer Center, Guangzhou, China; ^2^ Department of Radiology, Sun Yat-Sen University Cancer Center, Guangzhou, China; ^3^ Department of Pathology, Sun Yat-Sen University Cancer Center, Guangzhou, China; ^4^ Department of Molecular Diagnostics, Sun Yat-Sen University Cancer Center, Guangzhou, China

**Keywords:** epidermal growth factor receptor (EGFR), lung cancer, brain neoplasms, radiomics, magnetic resonance imaging

## Abstract

**Background:**

Lung cancer is the most common primary tumor metastasizing to the brain. A significant proportion of lung cancer patients show epidermal growth factor receptor (*EGFR*) mutation status discordance between the primary cancer and the corresponding brain metastases, which can affect prognosis and therapeutic decision-making. However, it is not always feasible to obtain brain metastases samples. The aim of this study was to establish a radiomic model to predict the *EGFR* mutation status of lung cancer brain metastases.

**Methods:**

Data from 162 patients with resected brain metastases originating from lung cancer (70 with mutant *EGFR*, 92 with wild-type *EGFR*) were retrospectively analyzed. Radiomic features were extracted using preoperative brain magnetic resonance (MR) images (contrast-enhanced T1-weighted imaging, T1CE; T2-weighted imaging, T2WI; T2 fluid-attenuated inversion recovery, T2 FLAIR; and combinations of these sequences), to establish machine learning-based models for predicting the *EGFR* status of excised brain metastases (108 metastases for training and 54 metastases for testing). The least absolute shrinkage selection operator was used to select informative features; radiomics models were built with logistic regression of the training cohort, and model performance was evaluated using an independent test set.

**Results:**

The best-performing model was a combination of 10 features selected from multiple sequences (two from T1CE, five from T2WI, and three from T2 FLAIR) in both the training and test sets, resulting in classification area under the curve, accuracy, sensitivity, and specificity values of 0.85 and 0.81, 77.8% and 75.9%, 83.7% and 73.1%, and 73.8% and 78.6%, respectively.

**Conclusions:**

Radiomic signatures integrating multi-sequence MR images have the potential to noninvasively predict the *EGFR* mutation status of lung cancer brain metastases.

## Introduction

Lung cancer patients frequently develop brain metastases (BMs), and these patients account for 51% of all BM patients (1). Epidermal growth factor receptor (*EGFR*) mutations are detected in 10%–60% of all non-small cell lung cancer (NSCLC) patients (2), and are associated with poor survival (3). Ligand binding to EGFR leads to receptor tyrosine kinase activation and mediates cell proliferation and invasion (4). Previous studies have shown that EGFR tyrosine kinase inhibitor treatment improves survival in patients with advanced NSCLC and sensitive *EGFR* mutations (5, 6). Thus, the determination of *EGFR* mutation status is critical for prognosis and treatment.

Discordance in *EGFR* status between primary lung tumors and BMs has been increasingly reported (7–9), indicating that it is not completely accurate to determine the *EGFR* status of BMs based on the status of the primary tumor. Therefore, molecular diagnostic tests are now recommended by clinical guidelines, to determine the eligibility of patients with advanced NSCLC for targeted therapies (10, 11). However, barriers remain to defining the *EGFR* status of BMs. First, magnetic resonance imaging (MRI) is the preferred method for BM screening, diagnosis, response evaluation, and follow-up, as radiologists can use it to depict the distribution and morphological characteristics of the BMs. However, MRI cannot be used to determine the molecular status of the BM. Second, obtaining BM materials by biopsy or resection may not be feasible depending on the patient’s status. Additionally, the risks of neurosurgery, sampling bias, and the fact that the procedure does not always provide an accurate account of the intrinsic intertumor and intratumor heterogeneity must be considered. These issues emphasize the need to develop an innovative approach for deriving biomarkers of metastasis. Radiomics is an emerging technology that extracts high-dimensional features from images to mine the potential biological characteristics of tumors. Studies have evaluated the relationship of radiomics features with the isocitrate dehydrogenase gene status of gliomas (12) or the *BRAF* gene status of melanoma BMs (13). Although several studies have applied radiomics to identify *EGFR* mutations in either BMs or primary lung cancers using brain MRI, the study populations were relatively small, especially for patients with *EGFR* mutations, or the *EGFR* mutation status of the BMs was determined based on the primary tumor status, rather than samples obtained from the BMs (14–23).

Therefore, the aim of this study was to establish a radiomic model *via* machine learning to predict the *EGFR* status of BMs confirmed by postoperative histopathology, using preoperative brain MRI sequences. We hypothesized that differential *EGFR* expression levels in BMs could be captured by radiomic signatures.

## Materials and Methods

### Study Patients

**Figure 1:**
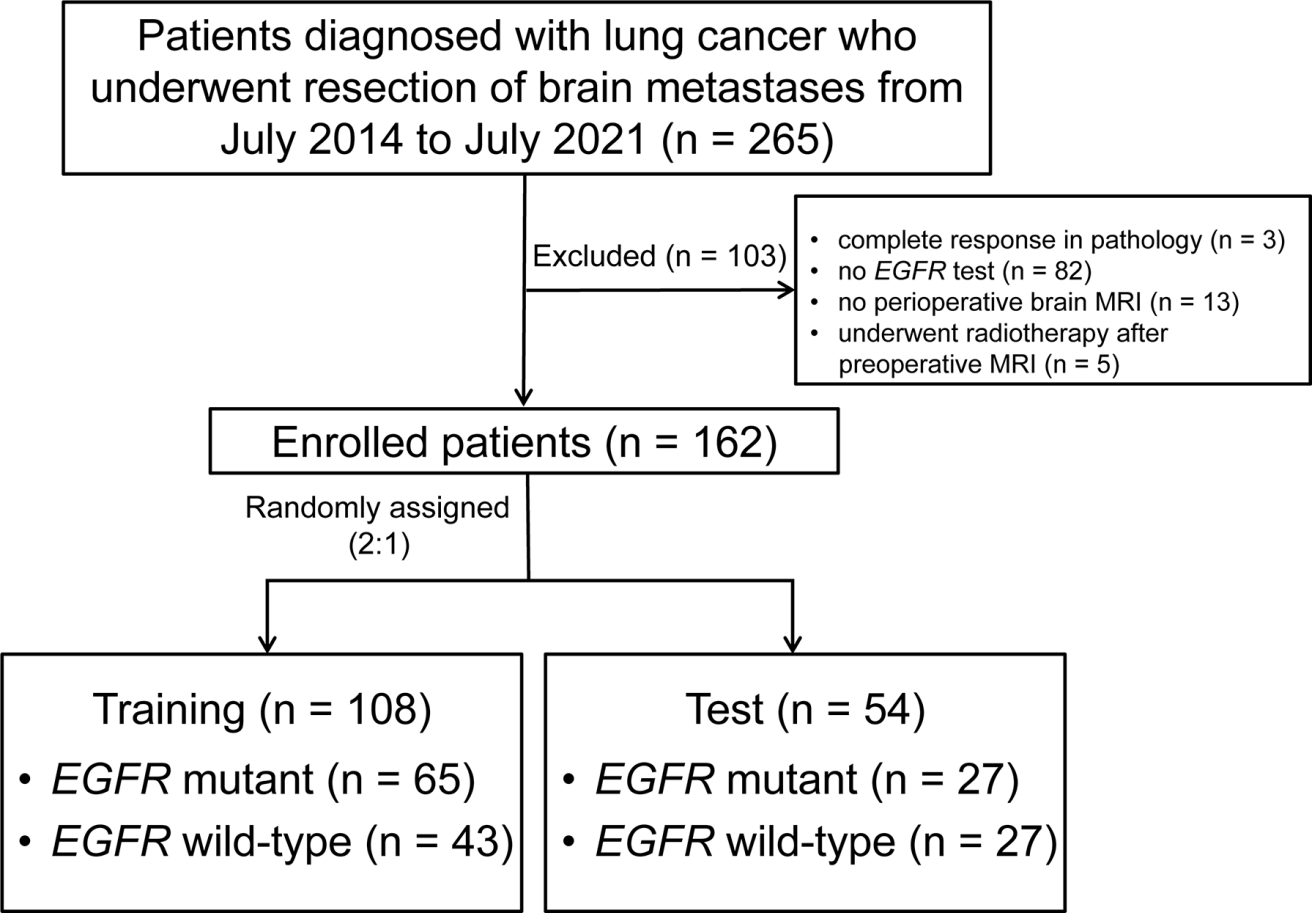
The participant recruitment process MRI, magnetic resonance imaging; *EGFR*, epidermal growth factor receptor.

This retrospective single-center study included patients with lung cancer who consecutively underwent BM surgical resection at Sun Yat-sen University Cancer Center from July 8, 2014, to July 6, 2021. Patients were included if they: (a) had primary lung cancer confirmed by biopsy or postoperative pathology, (b) had been diagnosed with BM, and (c) underwent surgical resection of the BM. Patients were excluded if they: (a) did not have complete pathology data for the BM, (b) did not receive an *EGFR* test for the excised BM, (c) did not undergo preoperative brain MRI, or (d) underwent brain radiotherapy during preoperative brain MRI and BM resection (**Figure 1**). There were no limitations on the number or size of the BMs. Clinical data (e.g., age, sex, and history of smoking) were acquired from the electronic medical records. This study was approved by the Institutional Review Board (No. B2021-198-01) of our center, and the requirement for informed consent was waived.

### Pathological Diagnosis and *EGFR* Testing

Histopathological sections of the primary lung cancer and the corresponding metastases were reviewed and classified according to the World Health Organization criteria by a pathologist with 8 years of experience (Y.J.Z.) (24). The mutation status in exons 18 to 21 of the *EGFR* gene was assessed using amplification-refractory mutation detection system–polymerase chain reaction or next-generation sequencing technology (25). The results were interpreted by a molecular diagnostician with 5 years of experience (Y.L.).

### Image Acquisition

Patients underwent brain MRI with 1.5-T or 3.0-T scanners produced by different manufacturers. Contrast-enhanced T1-weighted imaging (T1CE), T2-weighted imaging (T2WI), and T2 fluid-attenuated inversion recovery (T2-FLAIR) data were collected for feature extraction. For the T1CE sequence, the three-dimensional acquisition was routinely performed in the sagittal plane according to our department protocols. The scanner details and typical imaging parameters of the three targeted sequences are provided in the **Supplementary Material 1**. The MRI examination performed closest to brain surgery was selected. For patients with multiple BMs, only the lesions that matched both the surgical pathology and *EGFR* testing results were included in the radiomic analysis. To accurately assess the genetic status of the BMs, patients were excluded if more than two BMs were removed simultaneously and their *EGFR* testing results did not match.

### Image Segmentation

**Figure 2:**
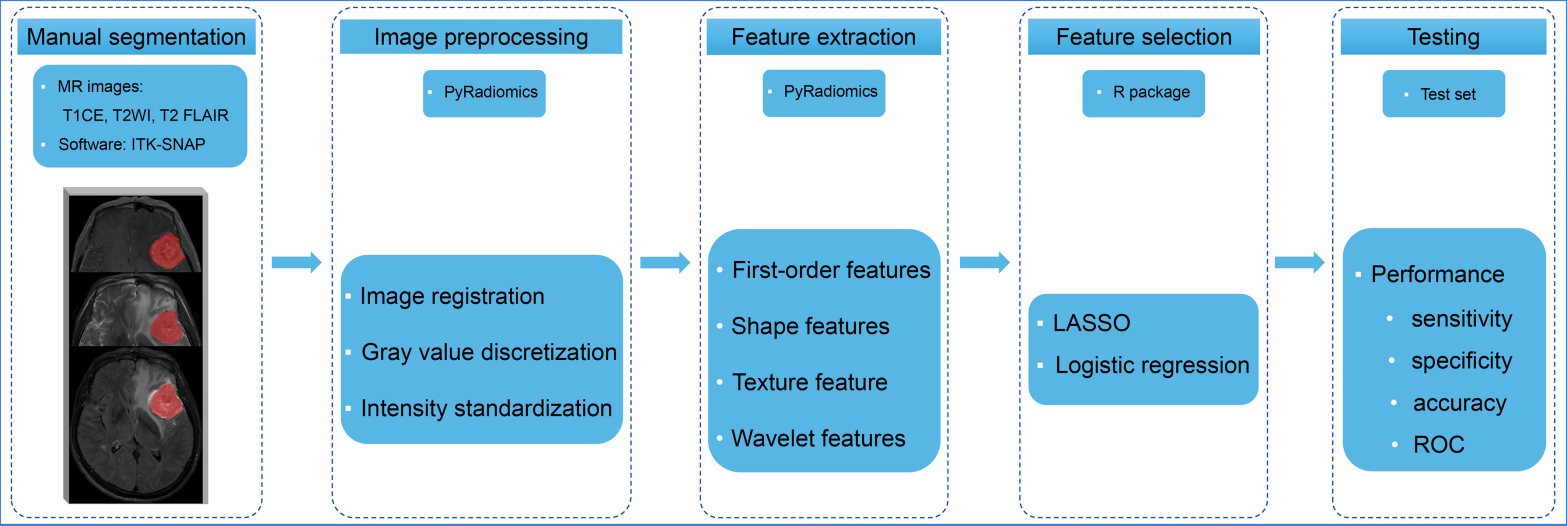
The radiomics analysis workflow Multiple-sequence MR images were selected and manually contoured. The radiomic features were extracted and selected from processed images to build models to predict the *EGFR* status of brain metastases. The performance of the models was evaluated using an independent test set. T1CE, contrast-enhanced T1-weighted imaging; T2WI, T2-weighted imaging; T2 FLAIR, T2 fluid-attenuated inversion recovery; LASSO, least absolute shrinkage and selection operator; ROC, receiver operating characteristic curve.

Radiomic analysis was performed as shown in **Figure 2**. Paired BMs imaged in the above three sequences were manually contoured around the lesions on a slice-by-slice basis in the axial view by a junior radiologist (L.X.) with 4 years of experience using ITK-SNAP (version 3.6; www.itksnap.org). The segmented regions of interest were confirmed by a senior neuroradiologist with 12 years of experience (Y.S.H.) and refined if necessary. To accurately match postoperative *EGFR* status with BMs in MR images, only the resected lesions were segmented for feature extraction.

### Radiomic Feature Extraction and Selection

Radiomic signatures were extracted using PyRadiomics, an open-source Python package for the extraction of radiomic features from medical images (http://www.radiomics.io/pyradiomics.html). This radiomic quantification platform enables the standardization of both image processing and feature definitions. Gray value discretization was performed with a fixed bin width of 25. Because MRI scanners with different field strengths were used, the intensity range of the images was normalized between 0 and 100 as a default set by the platform. We performed resampling with a pixel spacing of (3, 3, 3). The descriptions and feature explanations can be found on the PyRadiomics website. The parameter settings for image preprocessing and the feature extraction details are provided in **Supplementary Table S1**.

To obtain stable radiomic features for modeling and to evaluate the variability of these signatures, we randomly selected 40 patients from the cohort and their brain tumors were independently segmented by two radiologists (L.X. and Y.S.H.). The interclass correlation coefficient (ICC) was used to assess the stability of each feature. Intraobserver stability was calculated for each feature (**Supplementary Figure S1**). Stable radiomic features were defined as ICC values > 0.7. An initial selection was performed by deleting collinear strongly correlated variables detected using Pearson’s correlation analysis, for which the cut-off correlation coefficient value was 0.95. Univariate analysis was performed for each feature, and features with *P* < 0.05 were considered for selection. Marginally significant features were selected using the least absolute shrinkage and selection operator (LASSO) and a logistic regression model, which performed variable selection and regularization to enhance the prediction accuracy and interpretability of the statistical model. All features with non-zero coefficients were selected in this step. Finally, backward elimination was selectively performed to reduce the number of features included in the final set (**Supplementary Table S1**). The performance of the radiomic model was tested internally using an independent test cohort.

### Statistical Analysis

Patient characteristics were compared using a chi-square test for categorical variables, an independent Student’s t test for normally distributed continuous variables, and a Mann–Whitney U test for continuous variables without a normal distribution. The *EGFR* expression status in the primary cancers and BMs was calculated and compared using a Wilcoxon signed ranks test. We used the following R packages: irr (version 0.84.1) for calculating ICCs; caret (version 6.0–86) for Pearson’s correlation analyses; glmnet (version 4.0–2) for LASSO logistic regression analysis; rms (version 6.0–1) for logistic regression analysis; and pROC (version 1.17) for receiver operating characteristic curve (ROC) and area under the curve (AUC) analyses. The discrimination performance of the established model was quantified using ROC and AUC values, sensitivity, specificity, and accuracy. All statistical tests were two-sided, and a *P-*value < 0.05 was considered statistically significant. Statistical analyses were performed using R software version 4.0.2 (http://www.r-project.org/).

## Results

### Patient Characteristics

As shown in **Figure 1**, 265 patients with lung cancer BMs were enrolled in the study. One hundred and three patients were excluded due to a complete response revealed by postoperative pathology (n = 3), the absence of *EGFR* gene testing (n = 82), a lack of preoperative brain MRI (n = 13), or having undergone brain radiotherapy after preoperative MRI (n = 5). Thus, 162 patients were finally included.

All patients had a single BM removed. The median interval between MRI scanning and resection was 6 days (range, 0–75 days). Of the 162 patients (age, 57 ± 9 years [range, 22–74 years]; 97 [59.9%] males), 62 (38.2%) had a history of smoking, 133 (82.1%) were diagnosed with adenocarcinoma, 95 (58.6%) had a single BM, and 11 patients (6.8%) had more than 10 lesions. The distributions of patient and lesion characteristics in the training and test sets are provided in **Table 1**. There were no significant differences in baseline characteristics between the training and test sets.

**Table 1:** Patient and brain metastasis characteristics.

Characteristics	Training	Test	*P*
**No. of patients**	108	54	
**No. of male patients**	67 (62)	30 (56)	0.428
**Average age (years)**	57 ± 9	54 ± 10	0.427
**No. of smokers**	58 (54)	25 (46)	0.374
**Histology**			0.246
** adenocarcinoma**	86 (80)	47 (87)	
** non-adenocarcinoma**	22 (20)	7 (13)	
**No. of brain metastases**			0.354
** 1**	61 (56)	34 (63)	
** 2**	18 (17)	10 (19)	
** 3**	10 (9)	4 (7)	
** 4-10**	12 (11)	2 (4)	
** >10**	7 (6)	4 (7)	
**Excised brain metastases**			
** EGFR status**			
** mutation in exon**			
** 18**	2 (2)	2 (4)	0.333
** 19**	28 (26)	14 (26)	
** 20**	2 (2)	0	
** 21**	10 (9)	11 (20)	
** 20** & **21**	1(1)	0	
** wild-type**	65 (60)	27 (50)	
**Size (mm)**	40 ± 14	39 ± 13	0.577
**Location**			0.086
** cerebrum**	91 (84)	46 (85)	
** cerebellum**	14 (13)	8 (15)	
** brainstem**	1 (1)	0	
** lateral ventricle**	2 (2)	0	
**Cyst present**	92 (85)	44 (81)	0.545
**Hemorrhage present**	34 (31)	15 (28)	0.629
**Median time between the MRI and the resection (days)**	6	6	0.404

Data represent the number, number (%), or mean (standard deviation); EGFR, epidermal growth factor receptor, MRI, magnetic resonance imaging.

### Resected BM Characteristics

The targeted lesions had a mean diameter of 39 ± 14 mm (range, 13–76 mm), and most of them were located in the cerebrum (85%), followed by the cerebellum (17%). Cysts and hemorrhages were observed in 84% and 30% of the BMs, respectively.

Of the 162 resected BMs used for radiomics analysis, 70 (43.2%) were positive for an *EGFR* mutation and 92 (56.8%) were negative. The frequency of *EGFR* mutations was higher in patients with adenocarcinoma than in those with non-adenocarcinoma (adenocarcinoma vs. non-adenocarcinoma, 48.1% vs. 18.5%, *P* = 0.023). *EGFR* mutations were present at a significantly higher frequency in females than in males (64.6% vs. 28.9%, *P* < 0.001). None of the females had a history of smoking; thus, we analyzed the *EGFR* status in males and found a higher incidence of *EGFR* mutations in males with a history of smoking than those without (42.9% vs. 21.0%, *P =* 0.022). Of the patients with *EGFR* mutations, 42 had mutations in exon 19 (60.0%); 21 (30.0%) had mutations in exon 21; and 7 (10.0%) had rare mutations in exon 18 (including three with G719X missense mutations and one with an S768I-V769L compound mutation), an insertion mutation in exon 20 (S768I), and compound mutations in exons 20 (T790M) and 21 (L858R).

**Figure 3:**
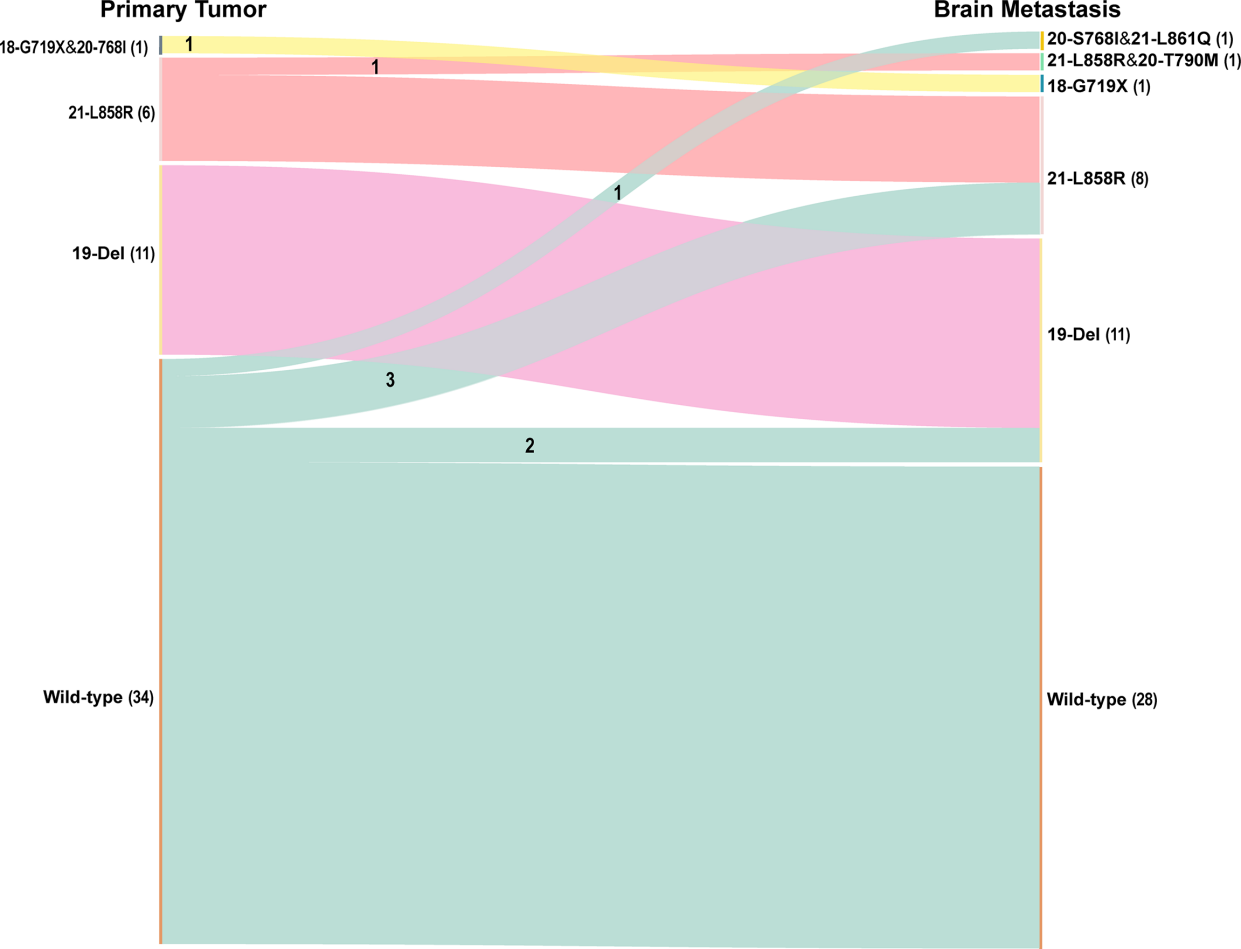
The *EGFR* mutation status distributions of primary lung cancers and paired metastases Overall, the *EGFR* status showed a discordance rate of 15.4% between the primary cancer and the matched brain metastases. The number of patients is provided in parentheses. *EGFR*, epidermal growth factor receptor.

Of the 265 patients initially included in the study, the *EGFR* mutation status of 52 patients was available for both the primary lung cancer and the corresponding BMs. An *EGFR* mutation was detected in 18 lung cancers and 24 BMs. Of the patients who had *EGFR* mutation-positive primary tumors, two (11.1%) had different mutations in the metastatic tumors. In one patient, there was a change from compound mutations in exons 18 and 21 to a mutation in exon 18, and in another patient, there was a change from a mutation in exon 21 to compound mutations in exons 18 and 21. No patients that were positive for an *EGFR* mutation in the primary tumor showed a loss of the mutation in the BM. Of the 34 patients who had *EGFR* mutation-negative primary tumors, 6 (17.6%) developed a new *EGFR* mutation in the metastatic tumor (two with deletion mutations in exon 19, three with missense mutations in exon 21, and one with co-current mutations in exons 20 and 21). We defined discordance as a conversion of mutation status from mutant to wild-type or vice versa or a change from one type of *EGFR* mutation to a different type. Thus, *EGFR* mutation status showed an overall discordance rate of 15.4% (Wilcoxon signed ranks test, *P* = 0.461) between the primary cancer and the corresponding BMs. The *EGFR* mutation status distributions are presented in **Figure 3**.

### Feature Selection and Radiomic Signature Construction

From each sequence, we extracted 1,470 radiomic features, comprising 14 shape features, 288 first-order features, 352 gray-level co-occurrence matrix features, 224 gray-level dependence matrix features, 256 gray-level run-length matrix features, 256 gray-level size-zone matrix features, and 80 neighboring gray-tone difference matrix features. Through a series of methods for selection (e.g., ICC, Pearson’s correlation, univariate analysis, LASSO, and backward elimination; **Supplementary Table S1**), the number of radiomic features selected to differentiate the *EGFR* mutation status was reduced to four, eight, four, and ten for T1CE, T2WI, T2 FLAIR, and combined sequences, respectively, to build the radiomic models. Half of the features in the combined model were from T2WI (5/10). **Table 2** lists the significant features used to differentiate *EGFR* mutation status in the various sequence models.

**Table 2:** Radiomic features used to differentiate *EGFR* mutation status in various sequences.

Sequences	Sequence	Feature category	Features
**Combination**			
	T1CE	Original shape	Flatness
	T1CE	Wavelet.HHH GLCM	Cluster shade
	T1CE	Square GLSZM	Low gray-level zone Emphasis
	T2WI	GLSZM	Low gray-level zone Emphasis
	T2WI	Wavelet.LHL GLCM	Correlation
	T2WI	Wavelet.HHH GLCM	Imc 2
	T2WI	Square root first order	Skewness
	T2WI	Exponential GLCM	Correlation
	T2 FLAIR	Wavelet.HLH GLSZM	Gray-level variance
	T2 FLAIR	Exponential first order	Interquartile range
**Single**			
	T1CE	Original shape	Flatness
	T1CE	First order	Median
	T1CE	GLCM	Cluster shade
	T1CE	GLSZM	Low gray-level zone Emphasis
	T2WI	Original shape	Elongation
	T2WI	GLSZM	Low gray-level zone Emphasis
	T2WI	Wavelet.LLH first order	10^th^ Percentile
	T2WI	Wavelet.LHL GLCM	Correlation
	T2WI	Wavelet.HHH GLCM	Imc 2
	T2WI	Square root first order	Skewness
	T2WI	Exponential GLCM	Correlation
	T2WI	Exponential GLSZM	Low gray-level zone Emphasis
	T2 FLAIR	GLCM	Correlation
	T2 FLAIR	Exponential first order	Interquartile range
	T2 FLAIR	Wavelet.HLH GLSZM	Gray-level variance
	T2 FLAIR	Gradient first order	Minimum

EGFR, epidermal growth factor receptor; T1CE, contrast-enhanced T1-weighted imaging; T2WI, T2-weighted imaging; T2-FLAIR, T2 fluid-attenuated inversion recovery; GLCM, gray-level co-occurrence matrix; GLSZM, gray-level size zone matrix.

### Prediction Performance

**Figure 4:**
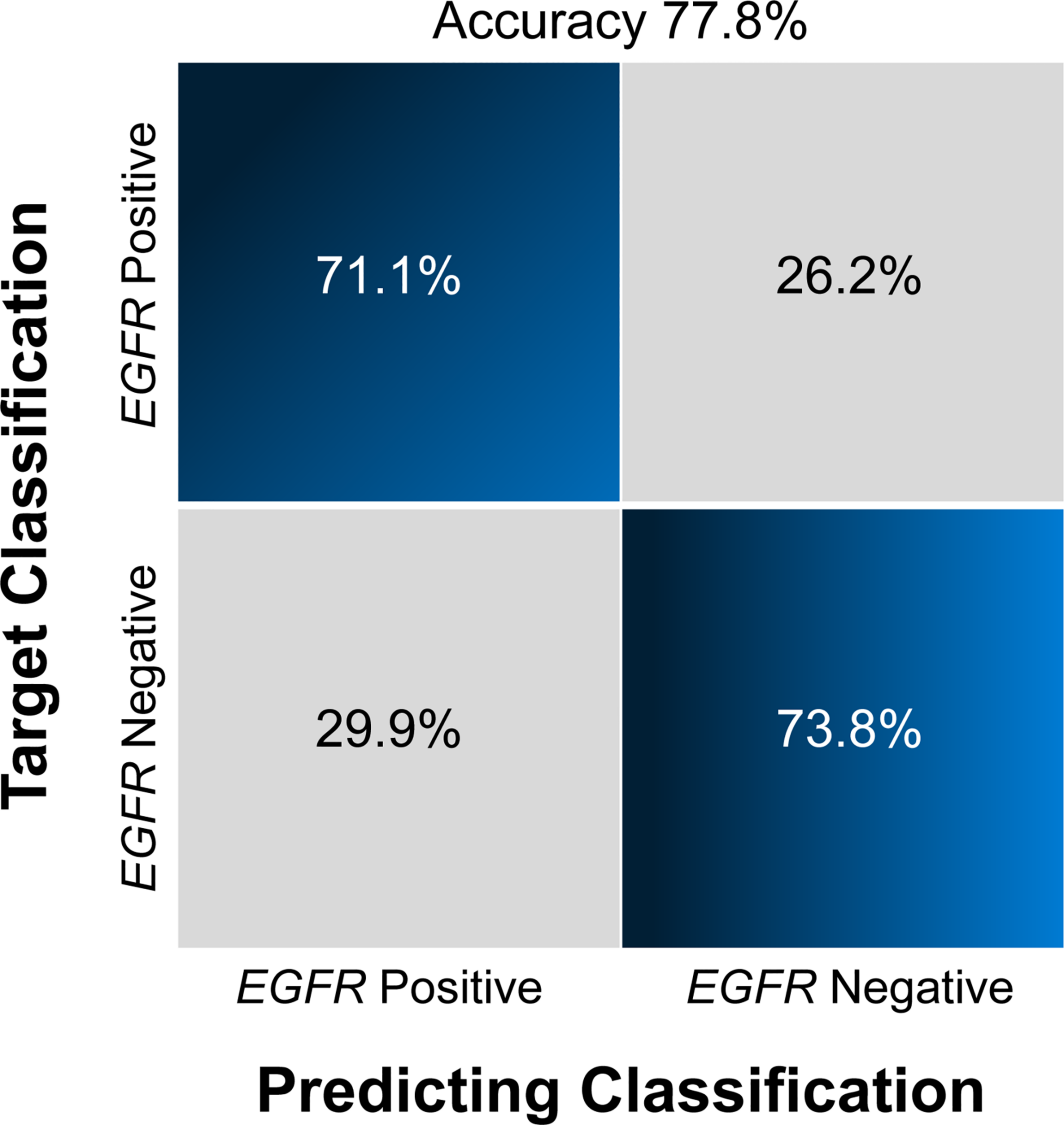
Confusion matrix **(A)** and ROCs **(B)** for the classification of *EGFR* mutation status in the test set The confusion matrix was generated using a combined model. The combined model appeared to achieve a higher AUC than any individual sequence, but the differences were not statistically significant. *EGFR*, epidermal growth factor receptor; ROC, receiver operating characteristics curve; AUC, area under the curve; T1CE, contrast-enhanced T1-weighted imaging; T2-FLAIR, T2 fluid-attenuated inversion recovery; T2WI, T2-weighted imaging; combination, combined model extracting features from the three sequences.

**Figure 5:**
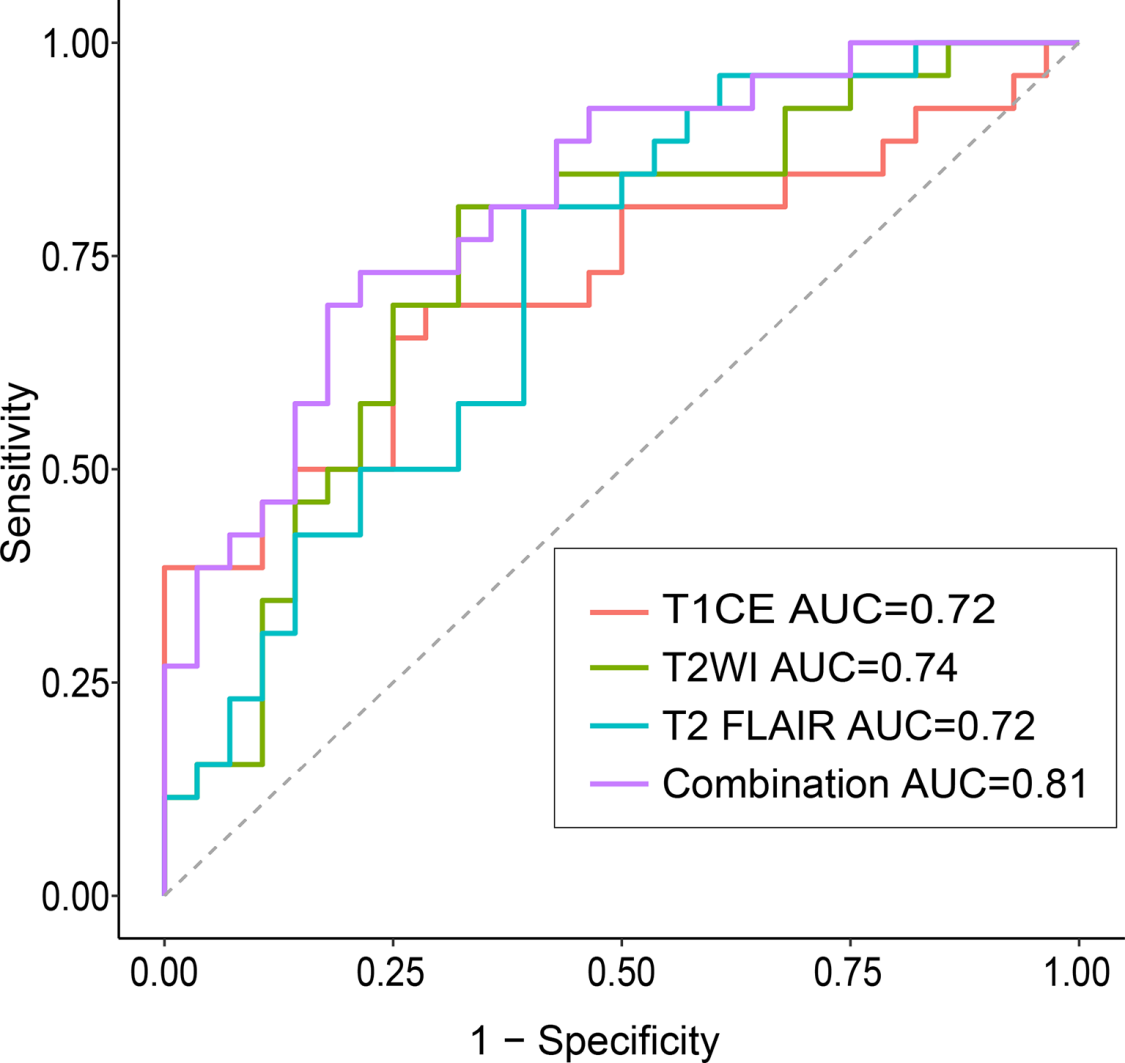
The decision curve analyses of various models The best decision benefit was observed with the combined model. T1CE, contrast-enhanced T1-weighted imaging; T2-FLAIR, T2 fluid-attenuated inversion recovery; T2WI, T2-weighted imaging; combination, combined model extracting features from three sequences.

For each MRI sequence, we built radiomic signatures using the training set and evaluated their classification performance in the test set. The prediction performance details are provided in **Table 3** , **Figures 4**,**5**.

**Table 3:** The performance of radiomics in predicting *EGFR* mutation status in various sequences.

Sequences	Sensitivity (95% CI)	Specificity (95% CI)	Accuracy (95% CI)	AUC (95% CI)	*P* ^a^
**Training**					
**Combination**	83.7 (72.7, 94.8)	73.8 (63.2, 84.5)	77.8 (77.5, 78.1)	0.85 (0.78, 0.92)	
**T1CE**	81.4 (69.8, 93.0)	56.9 (44.9, 69.0)	66.7 (66.3, 67.1)	0.74 (0.65, 0.84)	0.011^*^
**T2WI**	74.4 (61.4, 87.5)	65.6 (53.0, 76.2)	68.5 (68.1, 68.9)	0.76 (0.66, 0.85)	0.017^*^
**T2 FLAIR**	62.8 (48.3, 77.2)	69.2 (58.0, 80.5)	66.7 (66.3, 67.1)	0.69 (0.59, 0.79)	0.001^*^
**Test**					
**Combination**	73.1 (56.0, 90.1)	78.6 (63.4, 93.8)	75.9 (75.3, 76.6)	0.81 (0.70, 0.93)	
**T1CE**	69.2 (51.5,87.0)	71.4 (54.7, 88.2)	70.4 (69.6, 71.1)	0.72 (0.58, 0.86)	0.216
**T2WI**	80.8 (65.6, 95.9)	67.9 (50.6, 85.2)	74.1 (73.4, 74.8)	0.74 (0.61, 0.88)	0.182
**T2 FLAIR**	80.8 (65.6, 95.9)	60.7 (42.6, 78.8)	70.4 (69.6, 71.1)	0.72 (0.58, 0.86)	0.164

EGFR, epidermal growth factor receptor; AUC, area under the curve; CI, confidence interval; T1CE, contrast-enhanced T1-weighted imaging; T2-FLAIR, T2 fluid-attenuated inversion recovery; T2WI, T2-weighted imaging; ^a^, the AUC of T1CE, T2WI, and T2 FLAIR compared with the combination of the three sequences; *, statistically significant.

Overall, the combination sequences achieved the best AUC in both the training and test sets, with AUCs of 0.85 and 0.81, classification sensitivities of 83.7% and 73.1%, specificities of 73.8% and 75.9%, and accuracies of 77.8% and 75.9%, respectively. The AUCs were significantly different between the combination sequences and the single sequences in the training set (all *P* < 0.05), but showed no difference in the test set (*P* = 0.164–0.216). For single sequences, each sequence appeared to have a similar performance in the training and test sets, with AUC ranges of 0.69–0.76 and 0.72–0.74; classification sensitivities of 62.8%–81.4% and 69.2%–80.8%; specificities of 56.9%–69.2% and 60.7%–71.4%; and accuracies of 66.7%–68.5% and 70.4%–74.1%. The T2WI model achieved a higher AUC than the T1CE or T2 FLAIR model. **Figure 4A** illustrates the confusion matrix of the classification results obtained using the combined model in the test set. **Figures 4B**, **5** show the ROC curves and the decision curve analysis for the classification of *EGFR* mutations in all models.

## Discussion

In this proof-of-concept study, we extracted radiomic features from multiple MRI sequence images (T1CE, T2WI, and T2 FLAIR) of excised BMs originating from lung cancer and used these features to build machine-learning models for the classification of *EGFR* mutation status in BMs. Compared with a single sequence, the combination model, which extracted 10 key features from three sequences, achieved higher overall identification performance, yielding an AUC value of 0.81 in the independent test set. Additionally, the rate of discordance of *EGFR* mutation status between primary lung tumors and paired BMs was 15.4% in the 52 patients who underwent *EGFR* gene testing in both the primary tumor and the BM. Our findings indicate that the proposed radiomics signatures based on brain MRI can distinguish between mutant and wild-type *EGFR* in BMs, and the switch in *EGFR* status observed between the primary tumor and the BMs also indicates the importance of considering that the *EGFR* gene mutation status may differ between the metastases and the primary tumor.

New molecular agents targeting specific pathways have been developed and key molecules in tumor growth and progression have been identified. A typical example of such a target is the *EGFR* gene, which is an indicator of targeted treatment, an independent predictor of the treatment response, and a predictor of outcomes (26–28). Given the inconsistencies in target gene expression between primary tumors and their distant metastases, molecular diagnostic testing is now recommended for metastases in patients with advanced NSCLC whenever possible, to determine their eligibility for targeted therapies. Such assessments are recommended by the American Society of Clinical Oncology (29) and the European Association of Neuro-Oncology-European Society for Medical Oncology (10). Currently, however, it is not always practical to obtain a specimen of the BM by biopsy or surgery.

Therefore, several studies have used radiomics models to noninvasively predict the *EGFR* mutation status of lung cancer or BMs using brain MRI (15, 21). Ahn et al. extracted features from T1CE of 61 patients comprising 210 BMs with a size > 5 mm, and used several machine-learning algorithms to predict the *EGFR* gene mutation status of primary lung cancer, reaching an accuracy of 86.7% (AUC, 0.868) (15). In a similar study, Chen et al. built a model based on radiomic features generated by T1CE and T2 FLAIR (110 patients with 452 lesions, of whom 75 were *EGFR* positive) and clinical data using random forest classifiers, to classify *EGFR*, anaplastic lymphoma kinase, and Kirsten rat sarcoma virus gene mutation status in primary lung tumors and generated AUC values of 0.91, 0.92, and 0.99, respectively (21). However, both of these previous studies assumed an identical molecular profile in the BMs, thus overlooking possible discordances in *EGFR* mutation status between the lung cancer and the BMs. Additionally, there was no separate test set to validate the model performance, which may have led to overfitting.

Limited efforts have been focused on radiomics signatures to detect *EGFR* mutation status in BMs. Wang et al. analyzed four sequences (T1CE, T2WI, T2 FLAIR, and diffusion tensor images [DWI]) collected from 52 lung adenocarcinoma patients (28 with mutant *EGFR*, 24 with wild-type *EGFR*) (23). Although they concluded that the radiomics signature of T2 FLAIR achieved an AUC of 0.871, an accuracy of 0.845, a sensitivity of 0.901, and a specificity of 0.891 for discriminating *EGFR* mutation status using an independent testing data set, they also assumed that *EGFR* expression was consistent between the metastatic tumor and the primary tumor, which may not be accurate as discussed above. Haim et al. applied a deep-learning approach, using a ResNet-50 convolutional neural network, to predict *EGFR* mutation status in NSCLC BMs based on the *EGFR* testing results from resected BMs (20). However, they used data from a small cohort of 59 patients, of which only 16 patients were *EGFR*-positive. Moreover, they cropped regions of interest of the mid-tumor region and ± two slices for each patient. Such areas may be not sufficient to represent the entire tumor and may miss the three-dimensional features of the tumor. In contrast to previous studies, we enrolled, to the best of our knowledge, the largest reported study population of patients who underwent resection of their lung cancer BMs, to propose a radiomics signature based on multiple sequences of brain MRI. Moreover, despite adenocarcinomas showing the highest *EGFR* mutation rate among all histological cancer types, we included all patients with lung cancer, unlike other studies that exclusively selected patients with NSCLC or adenocarcinoma. Furthermore, we evaluated the *EGFR* mutation status in resected brain samples, which may better reflect the real mutation status. In addition, we used an open-source tool, Pyradiomics, for radiomics feature extraction, which may have improved the reproducibility of the feature extraction process.

We also found that the combination of features from multiple sequences had better classification performance than a single sequence, which was consistent with the study of Park et al. (18). Compared to single sequence, they reported that features extracted from the integration of T1CE and diffusion tensor images improved the capacity to determine the *EGFR* mutation status of BMs derived from lung cancer. Of the 10 features analyzed in our study, the biggest contribution came from T2WI. Furthermore, more second-order features than first-order features were selected, implying that multiparametric high-throughput characteristics enable a more accurate assessment than single parameters. Of the single sequences used to predict *EGFR* status, we found that the radiomic signatures of T2WI had the best performance. This differs from the result reported by Wang et al. (23), who found that T2-FLAIR yielded better *EGFR* mutation discrimination than TICE, T2WI, and DWI. Our results indicate that multiple sequences have higher predictive value than single sequences for the determination of *EGFR* mutation status.

Another finding in our study was that the discordance rate between the primary tumors and the corresponding BMs reached 15.4%. These results were comparable to those of previous studies that have reported heterogeneity in *EGFR* mutations between primary tumors and BMs, with variability rates ranging from 12% to 33% (8, 9, 30). Discordance between primary and metastatic tumors may be explained by clonal selection and intratumor heterogeneity (31). Clonal selection during the multistep metastatic process, combined with the potential effects of the tumor microenvironment and/or the treatment, may explain the discordance observed in metachronous metastases. Moreover, cancer is a highly heterogeneous disease, and polyclonal cell lines may exist with various *EGFR* statuses. Finally, the effect of different techniques on discordance cannot be excluded (32). Notably, two rare mutations were found in our study. A male patient with adenocarcinoma had both a deletion in exon 19 and an L858R missense mutation in exon 21 in the primary tumor, but the mutation in exon 21 was lost in the BM. Another adenocarcinoma in a female patient was found to have an S768I insertion in exon 20 and a G719X missense mutation in exon 18, but, similarly, the insertion was lost in the BM. The mechanism responsible for these changes will be investigated in future studies. We did not observe any *EGFR-*positive primary tumors that switched to an *EGFR-*negative form in BMs. Our data suggest that gaining *EGFR* mutations or switching *EGFR* subtypes may be more frequent than the complete loss of *EFGR* mutations when the primary tumors metastasize to the brain (negative to positive vs. positive to negative, 17.6% vs. 0%, Yates’ continuity correction, *P* = 0.567; change mutation type vs. positive to negative, 11.1% vs. 0%, Fisher’s exact test, *P* = 0.486), but these differences did not reach statistical significance, possibly due to the small number of samples.

This study has several limitations. First, this was a retrospective single-center design, which may have created selection bias. The performance of the model should be validated using a larger prospective multi-center dataset. Nonetheless, this is the largest reported cohort exploring the feasibility of classifying *EGFR* expression in BMs based on radiomics. Second, as in most previous studies, a region of interest was delineated for the entire metastasis. We did not analyze the subregional features of the tumor, e.g., the areas with enhancement, necrosis, hemorrhage, or edema. Third, more novel techniques such as deep learning or functional MRI were not applied to extract features. However, using an open-source Python package to extract features may have improved the reproducibility. In addition, conventional MRI sequences have wider adaptability in clinical practice. Finally, we did not distinguish between mutation subtypes, e.g., common vs. rare or sensitive vs. resistant mutations, given the limited number of samples with rare and resistant mutations.

## Conclusions

We demonstrated that it is feasible to apply a multi-sequence radiomic model to noninvasively predict the *EGFR* mutation status of lung cancer BMs. Moreover, the discordance observed between the primary tumors and the BMs indicates that *EGFR* alterations in metastases should be considered when a molecular targeted treatment is to be implemented.

## Data Availability Statement

The raw data supporting the conclusions of this article will be made available by the authors, without undue reservation.

## Ethics Statement

The studies involving human participants were reviewed and approved by Institutional Review Board of Sun Yat-Sen University Cancer Center, approval No. B2021-198-01. Written informed consent for participation was not required for this study in accordance with the national legislation and the institutional requirements.

## Author Contributions

Conceptualization: LZ, HX, XL, HL, CX. Literature search: XL. Study design: LZ, HX, XL, HL. Data curation: HX, XL, YZ, YL. Software: HX, XL. Supervision: HL, CX. Visualization: LZ, HX, XL. Writing – original draft: LZ. Editing: HX, XL. Review & approval: all authors

## Conflict of Interest

The authors declare that the research was conducted in the absence of any commercial or financial relationships that could be construed as a potential conflict of interest.

## Publisher’s Note

All claims expressed in this article are solely those of the authors and do not necessarily represent those of their affiliated organizations, or those of the publisher, the editors and the reviewers. Any product that may be evaluated in this article, or claim that may be made by its manufacturer, is not guaranteed or endorsed by the publisher.
