# Supplementary material for: Radiomic Signatures for Predicting EGFR Mutation Status in Lung Cancer Brain Metastases
Source: Front Oncol. 2022 Jul 14;12:931812. doi: 10.3389/fonc.2022.931812 (PMC9334014; doi:10.3389/fonc.2022.931812)
Supplement: Supplementary file 1 [file DataSheet_1.docx]

***Supplementary Material***

1. **Three sequences imaging parameters in three scanners** (1.5-T Signa HDxt, GE Healthcare; 3.0-T Discovery MR750w, GE Healthcare; 3.0-T Prisma, Siemens)

T1-CE sequences were acquired 5 min after contrast injection (MultiHance; Bracco Diagnostics, Princeton, NJ), and the scanning parameters were as follows (Signa HDxt/Discovery MR750w/Prisma): repetition time (TR), 633/170/250 ms; echo time (TE), 9/1,928/2.46 ms; field of view (FOV), 240 mm × 240 mm; voxel size, 0.63 mm × 1.1 mm/0.63 mm × 0.94 mm/0.63 mm × 1.03 mm; matrix, 384 × 224/384 × 256/384 × 234 ; slice thickness, 5 mm; slice gap, 1 mm.

T2WI imaging parameters were as follows: TR, 5,266/11,565/5,150 ms; TE, 92/79.8/99 ms; FOV, 240 mm × 240 mm; voxel size, 0.67 mm × 1.1 mm/0.63 mm × 0.63 mm/0.63 mm × 0.63 mm; matrix, 512 × 224/384 × 384//384 × 384; slice thickness, 5 mm; slice gap, 1 mm.

T2-FLAIR imaging parameters were as follows: TR, 8002/9000/8000 ms; TE, 126/89.5/98 ms; FOV, 240 mm × 240 mm; voxel size, 0.86 mm × 1.26 mm/0.75 mm × 0.94 mm/0.63 mm × 0.78 mm; matrix, 280 × 190/320 × 256/384 × 307; slice thickness, 5 mm; slice gap, 1 mm.

1. **Supplementary Figures**

**Figure S1 Interclass correlation coefficient for different sequences**

**
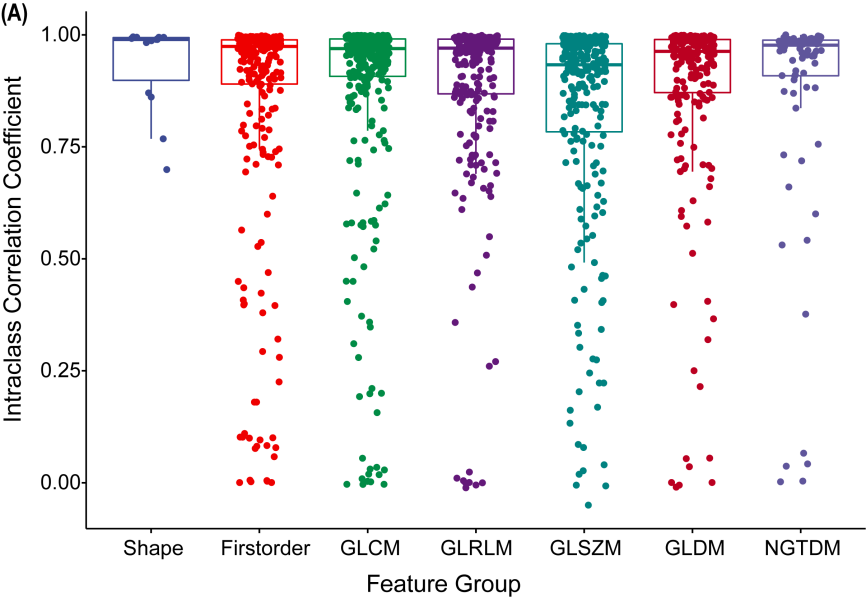
**

**
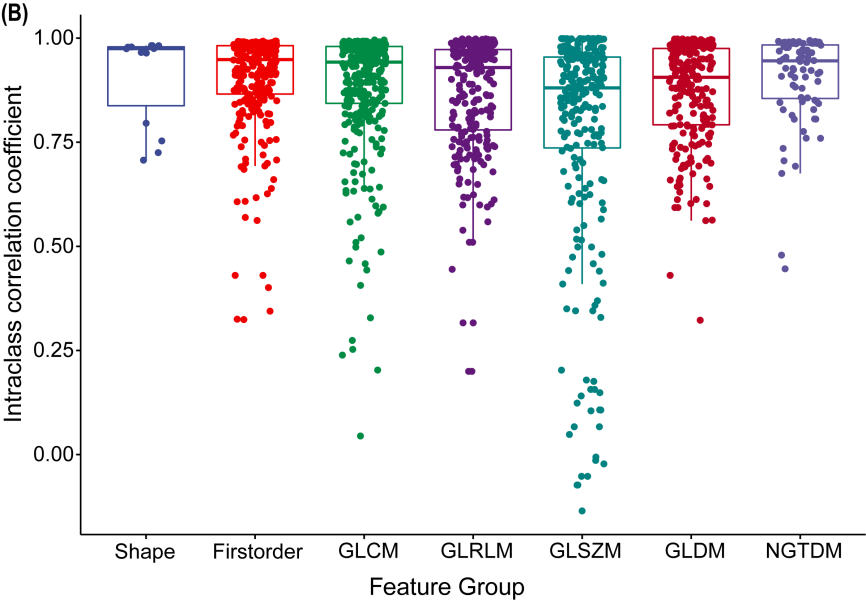
**

**
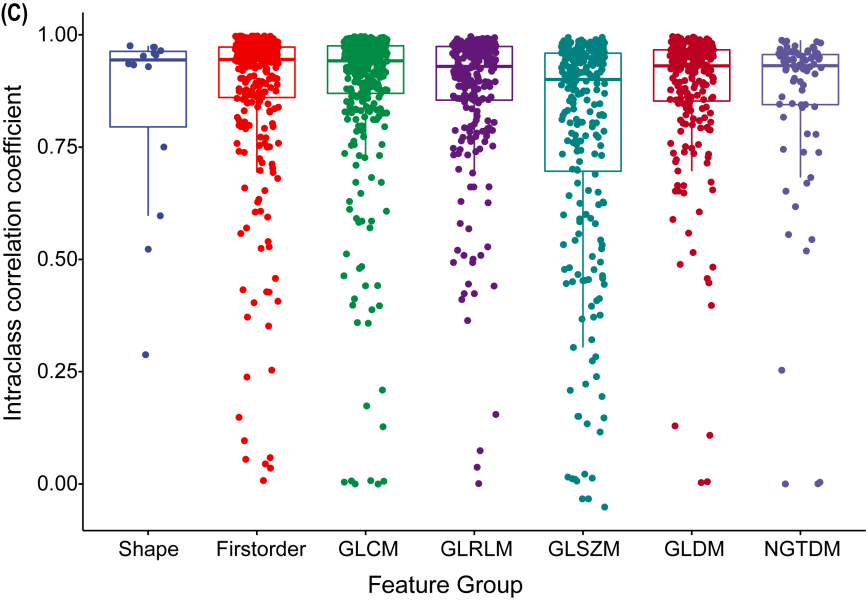
**

Figures (A), (B) and (C) were the ICCs for the frequences of contrast-enhanced T1-weighted imaging, T2-weighted imaging, and T2 fluid-attenuated inversion recovery, respectively. ICC = interclass correlation coefficient, glcm = gray level co-occurrence matrix, glrlm = gray level run length matrix, glszm = gray level size zone matrix, gldm = gray level dependence matrix, ngtdm = neighbouring gray tone difference matrix.

1. **Supplementary Table**

**Table S1** **Features extracted and selected for *EGFR* status radiomic signatures**

| Feature selection steps |  | MRI sequence | | | |
| --- | --- | --- | --- | --- | --- |
|  |  | T1CE | T2WI | T2 FLAIR | Combination^a^ |
| Total features extracted |  | 1,470 | 1,470 | 1,470 |  |
| ICC |  | 1,281 | 1,301 | 1,298 |  |
| Pearson correlation |  | 550 | 558 | 497 |  |
| Univariate analysis |  | 6 | 26 | 28 |  |
| LASSO |  | 4 | 12 | 12 |  |
| Backward elimination |  | 4 | 8 | 6 | 10^b^ |

a, combination of T1CE, T2WI and T2-FLAIR; b, the result of backward elimination from the T1CE (4 features), T2WI (8 features) and T2 FLAIR (6 features); *EGFR* = epidermal growth factor receptor, T1CE = contrast-enhanced T1-weighted imaging, T2-FLAIR = T2 fluid-attenuated inversion recovery, T2WI = T2-weighted imaging; ICC = interclass correlation coefficient, LASSO = least absolute shrinkage and selection operator
